# Maximum power extraction and DC-Bus voltage regulation in grid-connected PV/BES system using modified incremental inductance with a novel inverter control

**DOI:** 10.1038/s41598-022-22952-0

**Published:** 2022-11-19

**Authors:** Ibrahim AL-Wesabi, Fang Zhijian, Hassan M. Hussein Farh, Abdullrahman A. Al-Shamma’a, Hanlin Dong, Abdullah M. Al-Shaalan, Tarek Kandil

**Affiliations:** 1 School of Automation, China University of Geoscience, Wuhan, 430074 China; 2 Hubei Key Laboratory of Advanced Control and Intelligent Automation for Complex Systems, Wuhan, 430074 China; 3 Engineering Research Center of Intelligent Technology for Geo-Exploration, Ministry of Education, Wuhan, 430074 China; 4 Electrical Engineering Department, College of Engineering, Imam Mohammad Ibn Saud Islamic University, Riyadh, Saudi Arabia; 5 Electrical Engineering Department, College of Engineering, King Saud University, Riyadh, Saudi Arabia; 6 School of Engineering and Technology, Western Carolina University, Cullowhee, NC 28723 USA

**Keywords:** Electrical and electronic engineering, Renewable energy

## Abstract

Low ripples and variations in the DC-Bus voltage in single-phase Photovoltaic/Battery Energy Storage (PV/BES) grid-connected systems may cause significant harmonics distortion, instability, and reduction in power factor. The use of short-life electrolytic capacitor on the DC-Bus is considered a standard way for reducing these ripples and variations because of its large capacitance but results in short lifetime of the inverter. Replacing large electrolytic capacitors with small film capacitors can extend the lifetime of a PV/BES grid-connected system because small film capacitors have longer lifetime than large electrolytic capacitors. These film capacitors have low capacitance, which causes severe oscillations in the output current, and voltage drop due to huge ripples on the DC-Bus voltage. In this research, the main goal is to eliminate the output current ripples and voltage fluctuations associated with employing film capacitors. First, a modified incremental conductance (MIC) technique is proposed for tracking the maximum power by controlling the duty ratio of the DC-DC boost converter. Second, for the first time, a simple and novel d-q current regulation technique, which employs flowchart decision logic, is used in the DC-Bus control system for both the PV power system and the state of charge (SOC) of the BES. In this case, the DC-Bus controller is characterized by a cost-effective implementation because of its low sampling frequency. Although the presented approaches are successful in eliminating voltage distortion and fluctuations, they have unacceptable dynamic performance. Therefore, to improve the dynamic performance, BES was used to maintain a reliable and stable harvest from PV modules for varying loads while also increasing the dynamic performance of the overall system. The proposed PV/BES grid-connected systems, which employs a small 10-µF bus capacitor, is simulated and connected to the grid (230 V, 50 Hz). The DC-Bus voltage overshoot, undershoot and the total harmonics distortion (THD) of the output current for the proposed MIC are (1 V), (2.5 V) and (less than 5%), respectively. The average time response under rising radiation to track the global peak for MIC, traditional incremental conductance and variable step size incremental conductance are 1.403 s, 1.501 s and 1.113 s respectively. The obtained findings demonstrated the efficacy and superiority of the proposed d-q current control and MIC technique.

## Introduction

In comparison to previous photovoltaic (PV) architectures^1^, the PV/BES grid-connected system is more useful since each component is optimized for optimal power generation, they are low cost and quick to install (known as "Plug and Play"), and have enhanced flexibility and modularity^2^. The two-stage arrangement with a DC-Bus represents the standard configuration for PV/BES grid connected systems^3–8^.

Two obvious difficulties associated with the DC-Bus in the single-phase PV/BES grid-connected systems which are the frequency ripples of the DC-Bus voltage and overshoots and/or undershoots in bus control loop^9^. The ripples generally distort significantly the current reference with a visible distortion which is almost entirely a third harmonic and a phase shift. When voltage oscillations occur because of abrupt changes in input power, the safety mechanism is triggered, causing the input/output stage to be separated and the efficacy to be reduced. The control design’s goal is to reduce DC-Bus voltage fluctuations and output current ripples^10–14^.

The AC-driven (PWM) inverters are power converters that convert DC-Bus voltage to AC voltage. The PWM inverter's DC-Bus capacitor functions as an energy barrier to stabilize and keep the DC-Bus voltage at a relatively constant level. Therefore, the large capacitance of the electrolytic capacitors is often used as DC-Bus capacitors. Due to the limited life expectancy of the inverter, the high capacity of the electrolytic capacitor in the DC-Bus has an impact on the reliability of the inverter. In addition, when using a three-phase passive rectifier, excessive DC-Bus capacitance results in an increase in the total harmonic distortion (THD) of the input supply current such as a three-phase diode rectifier. As a result, a lot of effort was put into replacing electrolytic capacitors with small film capacitors that have a longer lifetime^15,16^.

Nevertheless, if the power inductance is important compared to the DC-Bus capacitor, the DC-Bus voltage of the inverter may get unbalanced, resulting in an overvoltage or undervoltage. Failure owing to the reduced DC-Bus capacitance may occur. As the constant power load has a negative dynamic impedance characteristics, this problem becomes a quite significant when the inverter supplies high power to it^17,18^. Because of this negative impedance feature, when the DC-Bus voltage increases and the DC-Bus current decreases, ensuring that the load's power remains constant. On the DC-Bus, this response might result in overvoltage or undervoltage. Negative impedance features can be avoided by creating an input filter whose parameters match the stability requirement^17^. If the filter settings are fixed, passive damping resistors can be used to modify the impedance of the system. Passive damping resistors, on the other hand, always add to the system's losses.

In^17,18^, the fluctuating DC-Bus voltage was actively stabilized by modifying the DC-Bus dynamic impedance to be positive. Since the output power is related to the output current, modifying the current command in^19^ may be used to change impedance. If the DC-Bus voltage oscillation frequency exceeds the current control bandwidth, voltage commands are revised rather than the current commands^17,18^. These techniques, on the other hand, presume that the input source voltage is constant DC while using linearized control law. This presumption results in the inverter's dynamic impedance to be dependent on the variation of the DC-Bus voltage since the changed impedance is exactly proportional to the DC-Bus voltage. Furthermore, under the control legislation, the technique in^17^ mandates the usage of load impedance.

Speedy load changes can potentially cause the DC-Bus to overvoltage or undervoltage^20^. The DC-Bus voltage will reduce the substantially if the output power is raised in steps, for example, since the energy stored in the capacitor is inadequate to maintain the DC-Bus voltage. The inverter response or control bandwidth) must be minimized enough to eliminate this short DC-Bus voltage fluctuation and keep it within a tolerable range. The overall performance will suffer because of the restricted control bandwidth.

**Figure 1:**
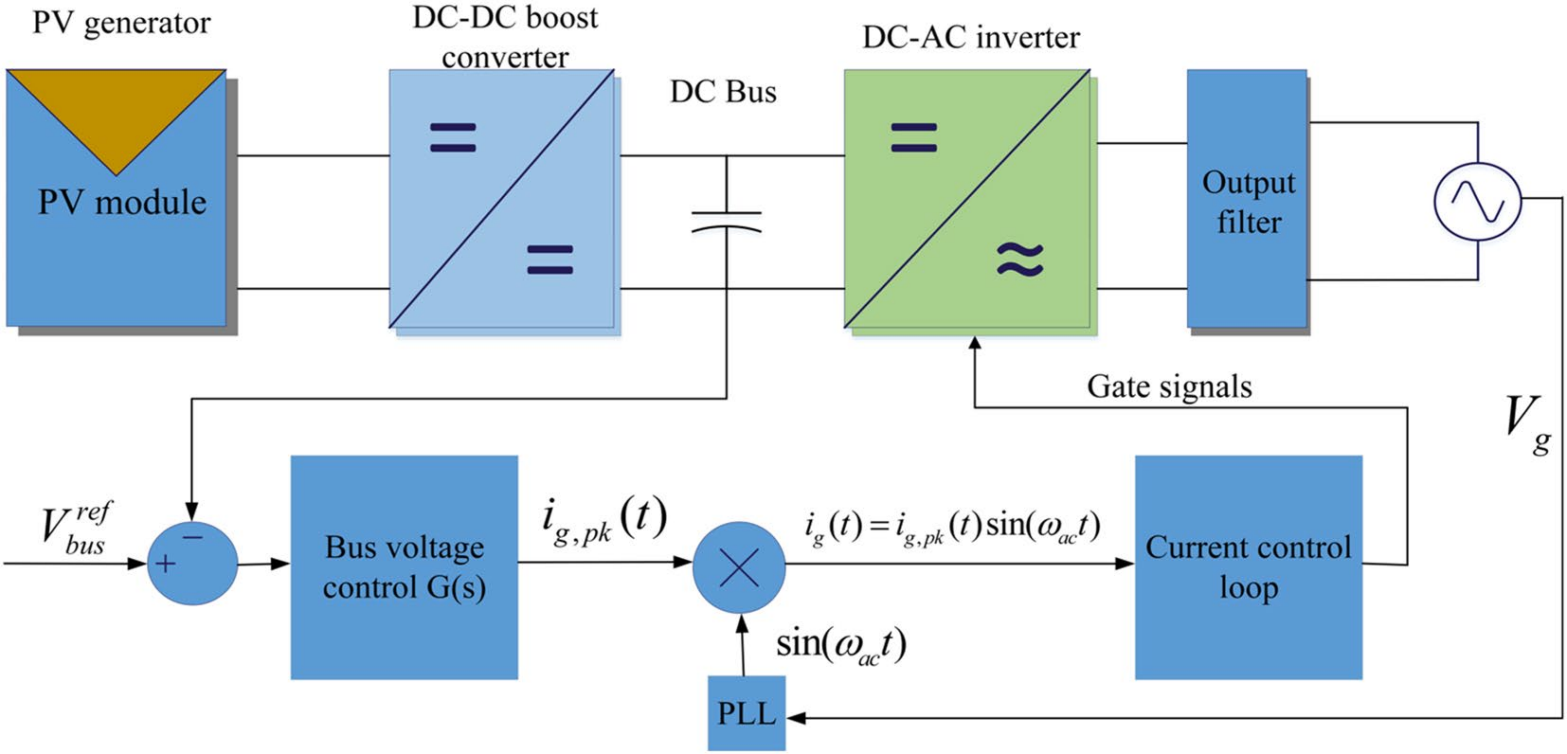
A single phase PV/BES grid-connected system with a common DC-Bus control mechanism.

Figure 1 depicts a 1-ph PV/BES grid-connected system with a common bus control system. To establish the output current reference, the difference between the DC-Bus voltage and the reference voltage is fed to the bus voltage controller and multiplied by the phase-locked loop (PLL) output signal. PWM signals are generated by this reference, which are utilized to operate the inverter via the current controller.

On the other hand, maximizing the PV power is always going to be challenging. To optimize PV power, researchers have proposed a variety of MPPT algorithms, including Fractional short-circuit current (FSCC), Fractional open-circuit voltage (FOCV), fuzzy logic (FL), neural network (NN), perturb and observe (P&O), and incremental conductance (IC)^21,22^. The simplest maximum power point tracking (MPPT) algorithms FSCC and FOCV are relying on the linearity of short-circuit current or open-circuit voltage to the MPP’s current or voltage. However, the PV panel is isolated using these methods, nevertheless, in order to detect the voltage or short-circuit current. Consequently, the periodic isolation of the panel causes an increase in energy loss^23^. As an alternative, fuzzy logic and neural networks are able to handle the nonlinearity of the PV panel and produce a consistent MPPT approach. The efficiency of this approach heavily depends on selecting the proper error calculation and a suitable rule foundation, which is fuzzy logic's fundamental weakness^24^. The data required for the training process for each PV array and location must be specifically gathered, and as PV features vary over time, the neural network must be trained on a regular basis^24^. Neural networks also have a number of other drawbacks. P&O and IC, on the other hand, are often utilized. These methods make use of the PV panel's feature. When the MPP is identified for P&O, steady-state oscillations are developed because of the disturbance caused by this approach to keep the MPP, which in turn causes an increase in power loss^25^. For IC, it is supported by the fact that the maximum power has a slope of P–V characteristic of zero, and theoretically, there is no disturbance once the MPP is discovered. As a result, oscillations are reduced. Due to a digital processing issue called truncation, the zero value on the slope of the P–V characteristic is seldom ever observed during implementation. As a result, when the irradiation is abruptly increased, the IC method may provide an erroneous response^26^. As a result, this study also intends to develop and put into practice a modified MIC algorithm that can correct the incorrect reaction that the traditional incremental conductance (TIC) algorithm makes when the irradiance is quickly raised. In order to identify the rise in solar irradiation, this article suggests a novel method. Instead of using the slope of the P-V characteristic, the fluctuation of voltage and current are employed to detect the increase in irradiation. The modified algorithm recognizes the rise in irradiance and renders an accurate judgment. Additionally, a small error is permitted in order to ensure that the slope is almost zero and to reduce steady-state oscillations.

On the other hand, this research proposed a novel DC-Bus voltage stabilizing technique under small capacitor. The stabilization is essentially based on the d-q current control for a single-phase inverter using flowchart decision logic for both the PV power system and state of charge (SOC) of battery storage system. The reason behind this is to ensure smooth transfer of power from the DC part to the AC part and vice versa as well as eliminate the effect of (grid) source voltage variation, both the estimated PV power system and SOC are used in the control law. Additionally, to eliminate the transient DC-Bus voltage ripples to an acceptable range, a simple DC-Bus voltage regulation method is also proposed based on a modified incremental conductance (MIC). Finally, BES was used to maintain a reliable and stable harvest from PV modules for loads while also improving the entire generation system’s dynamic performance. With the proposed method, the inverter can be kept operating with reduced DC-Bus capacitor.

The major contributions of this research are summarized as follows:

1. Proposed a simple new control method for d-q current control for a single-phase PV/BES grid-connected system using flow chart decision logic for both the PV power system and SOC of BES to ensure smooth power transfer from the DC part to the AC part and vice versa.
2. Applied MIC technique for tracking the maximum power point through controlling the duty ratio of the DC-DC boost converter to improve the efficiency of the hybrid PV/BES energy system and suppress the ripples in DC-bus voltage under rapid changing environmental condition.
3. For validation purpose, the proposed MIC algorithm was compared with variable step size incremental conductance (VSZIC), TIC algorithm and meta-heuristic algorithms (i.e. cuckoo search (CS), grey wolf optimization (GWO) particle swarm optimization (PSO) and water cycle algorithm (WCA)) as well as P&O method for tracking the global power under several different irradiance and temperature patterns.
4. BES used to maintain a reliable and stable output power generated from the PV systems for varying loads while also improving the entire system’s dynamic performance.

## Effects of reduced DC-Bus capacitor

In standalone and grid-connected PV structures, DC-Bus capacitor is the extremely important passive component. Harmonics and power factor reduction occur in single-phase PV inverters because the DC bus voltage exhibits a double frequency ripple. In order to reduce this ripple, large electrolytic capacitors, which have short lifetimes, are often used at the DC bus. To increase the lifetime of inverters, it is necessary to replace electrolytic capacitors with thin film capacitors, which have long life and low capacitance. This will result in three important outcomes: (1) size reduction, (2) cost reduction , and (3) dependability increase. The proposed high-power film capacitor may therefore be used to integrate the PV and inverter into a grid-connected PV system, decreasing the number of cables and their related losses and costs. On other hand, if the DC-Bus capacitor is very small, bus voltage overshoots will be large and unacceptable. There are three problems of using small DC-bus capacitor which has a low capacitance in PV/battery double stage single phase grid system which can be divided into three problems: instability of DC-bus voltage, output low-frequency ripple and system’s dynamic performance problem. Therefore, the main objective of this research is to overcome these three problems through using small DC-bus capacitor. In addition, a 10μF was used in our system because it is less than the values used in the literature. Therefore, a suitable DC-link capacitor size should be selected accurately to restrict the voltage ripple with in a permissible limit and to ensure a better and reliable system performance and to achieve high power density of the system.

### Instability analysis due to reduced DC-Bus capacitor

**Figure 2:**
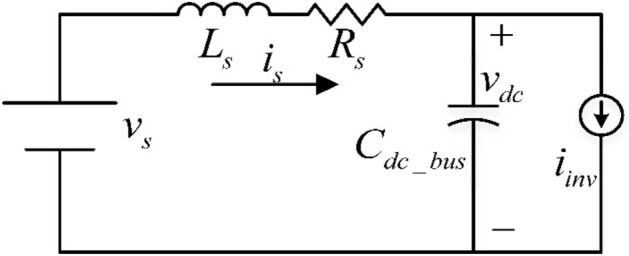
Source/Inverter/Load equivalent model.

Figure 2 shows the system model, which includes the source, inverter, and load but does not include the inverter’s PWM. A current source ($i_{inv}$) is used to simulate the inverter and load. The analogous source impedances are $L_{s}$ and $R_{s}$, respectively, while the DC-Bus capacitance is $C_{dc\_bus}$. The system’s dynamic equations are described by the following.

1 $$ L_{s} \frac{{di_{s} }}{dt} = v_{s} - R_{s} i_{s} - v_{dc}$$

2 $$C_{dc\_bus} \frac{{dv_{dc} }}{dt} = i_{s} - i_{inv}$$

If the load power is considered constant as $P_{L}$ , the inverter output current,${ }i_{inv}$ , can be indicated as

3 $$i_{inv} = \frac{{P_{L} }}{{v_{dc} }} = \frac{{P_{L} }}{{v_{dc0} + \tilde{v}_{dc} }}$$

where $v_{dc0}$ is the DC-Bus voltage’s mean value, and $\tilde{v}_{dc}$ is a DC-Bus voltage variation. If $\tilde{v}_{dc}$ is comparatively small to its mean value, the inverter output current can be linearized as Eq. (4).

4 $$i_{inv} = \frac{{P_{L} }}{{v_{dc0} + \tilde{v}_{dc} }} \cong \frac{{P_{L} }}{{v_{dc0} }} - \frac{{P_{L} }}{{v_{dc0}^{2} }}\tilde{v}_{dc}$$

The constant power demand works as a negative impedance against DC-Bus voltage change, as indicated in Eq. (4).

The dynamic Eq. (1) and Eq. (2) equations are the feature equations in the linearized inverter current.

5 $$s^{2} + (\frac{{R_{s} }}{{L_{s} }} - \frac{{P_{L} }}{{C_{dc\_bus} v_{dc0}^{2} }})s + (\frac{{v_{dc0}^{2} - R_{s} P_{L} }}{{L_{s} C_{dc\_bus} v_{dc0}^{2} }}) = 0$$

The DC-Bus capacitor's stability requirement may be calculated using Eq. (5).

6 $$C_{dc\_bus} > \frac{{L_{s} P_{L} }}{{R_{s} v_{dc0}^{2} }}$$

As a result, if the inverter’s capacitance fails to meet the principle in Eq. (6), the system conditions, with the DC-Bus voltage, become unbalanced or fluctuating. As a result, overvoltage breakdown can ruin semiconductor switches such as IGBTs.

In this study, the maximum permitted voltage ripple and input dc voltage are used to determine the size of the DC-Bus capacitor. It is observed that when the DC input voltage rises, the capacitance decreases, increasing the voltage ripple. Therefore, it is important to select a proper DC-Bus capacitor size to keep the voltage ripple within a reasonable range, to provide better and more reliable system operation, and to maximize the system's power density. Additionally, the grid-connected inverter selected for our research has a 10µF capacitor size according to the calculation. This study also discusses the various types of capacitors used as DC-Bus, along with their benefits and drawbacks. Equation (7) demonstrates that the nominal DC-Bus voltage and voltage ripple have an inverse relationship with the capacitance of the DC-Bus capacitor. As a result, a higher DC-Bus voltage results in a capacitor with a smaller capacitane which in turn has a higher voltage ripple. If the voltage ripple is not correctly managed, it might impair the PV generator's maximum power point functioning. Typically, to get the best output of a PV generator, the magnitude of voltage ripple should be kept around 5%.

7 $$C_{dc\_bus} = \frac{S}{{2\omega_{ac} V_{dc} + \tilde{v}_{dc} }}$$

### The trade-off between total harmonic distortion and fluctuations in the DC-Bus

For simplicity, the power stages are considered perfect in this section, and losses are ignored. According to the functioning of the common control loop in the DC-Bus that is represented in Fig. 1, the grid output current, *i*_*g*_, can be determined in Eq. (8).

8 $$i_{g} = i_{g,pk} (t)\sin (\omega_{ac} t)$$

According to the power balancing, the frequency ripple’s peak-to-peak value which appears on the DC-Bus voltage can be calculated using Eq.(8)^2,11^.

9 $$\Delta V_{P - P} = \frac{{P_{PV} }}{{\omega_{ac} V_{dc\_bus}^{ref} C_{dc\_bus} }}$$

where $P_{PV}$ is the input power generated, $\omega_{ac}$ is the grid frequency, $V_{dc\_bus}^{ref}$ is the average DC-Bus voltage, and $C_{dc\_bus}$ is the DC-Bus capacitance. The DC-Bus voltage, *v*_*bus*_, can then be approximated by Eq. (10).

10 $$v_{bus} (t) \approx V_{dc\_bus}^{ref} + \frac{{P_{PV} }}{{2\omega_{ac} V_{dc\_bus}^{ref} C_{dc\_bus} }}\sin (2\omega_{ac} t)$$

The DC-Bus voltage’s double frequency term is then fed to the bus control loop which results in a steady-state grid peak current as:

11 $$i_{g,pk} (t) = I_{g,pk} + \left| {G(j2\omega_{ac} )} \right|.\frac{{P_{PV} }}{{2\omega_{ac} V_{dc\_bus}^{ref} C_{dc\_bus} }}\sin (2\omega_{ac} t)$$

where |*G*(*j*2 $\omega_{ac}$)| is the gain of DC-Bus control loop in the second harmonic frequency, and *I*_*g,pk*_ is the grid output current’s average amplitude, which possibly expressed in terms of grid voltage’s amplitude (*V*_*g,pk*_) and input or output power in the preceding equation. The grid output current can be indicated from Eq. (12)^11,13^ by substituting Eq. (11) into Eq. (8) and utilizing trigonometric formulae.

12 $$i_{g} (t) = \left( {\sqrt {\left( {\frac{{2P_{PV} }}{{V_{g.pk} }}} \right)^{2} + \left( {\frac{{P_{PV} \left| {G(j2\omega_{ac} )} \right|}}{{4\omega_{ac} V_{dc\_bus}^{ref} C_{dc\_bus} }}} \right)^{2} } } \right)\sin (2\omega_{ac} t) + \frac{{P_{PV} \left| {G(j2\omega_{ac} )} \right|}}{{4\omega_{ac} V_{dc\_bus}^{ref} C_{dc\_bus} }}\cos (3\omega_{ac} t)$$

In the grid single-phase applications, the output current’s third harmonic is dominant, and the other harmonics can be ignored. As a result, Eq. (13) can be used to compute the output current THD^11,13^.

13 $$THD = \frac{{P_{PV} \left| {G(j2\omega_{ac} )} \right|}}{{4\omega_{ac} V_{dc\_bus}^{ref} C_{dc\_bus} }} \times \left( {\left( {\frac{{2P_{PV} }}{{V_{g.pk} }}} \right)^{2} + \left( {\frac{{P_{PV} \left| {G(j2\omega_{ac} )} \right|}}{{4\omega_{ac} V_{dc\_bus}^{ref} C_{dc\_bus} }}} \right)^{2} } \right)^{ - 0.5}$$

The Taylor series-based linear approximation of Eq. (13) is presented in Eq. (14) and is accurate for THD values less than 20%^11,13^.

14 $$THD \approx \left( {\frac{{V_{g,pk} }}{{8\omega_{ac} V_{dc\_bus}^{ref} }}} \right).\frac{{\left| {G(j2\omega_{ac} )} \right|}}{{C_{dc\_bus} }}$$

As the gain increases, the DC-Bus control loop becomes faster which minimizes the DC-Bus voltage/over/undershoot in the transient response while increasing the output current’s THD at steady-state^11,13^. In Eq. (14) there is a trade-off in the steady-state response between bus voltage over/undershoots and THD of the output current in the transient response. The use of a standard PI controller as the DC-Bus controller has the straightforward consequence that it is impossible to lower these two values at the same time^11,13^.

## Control of power converters

**Figure 3:**
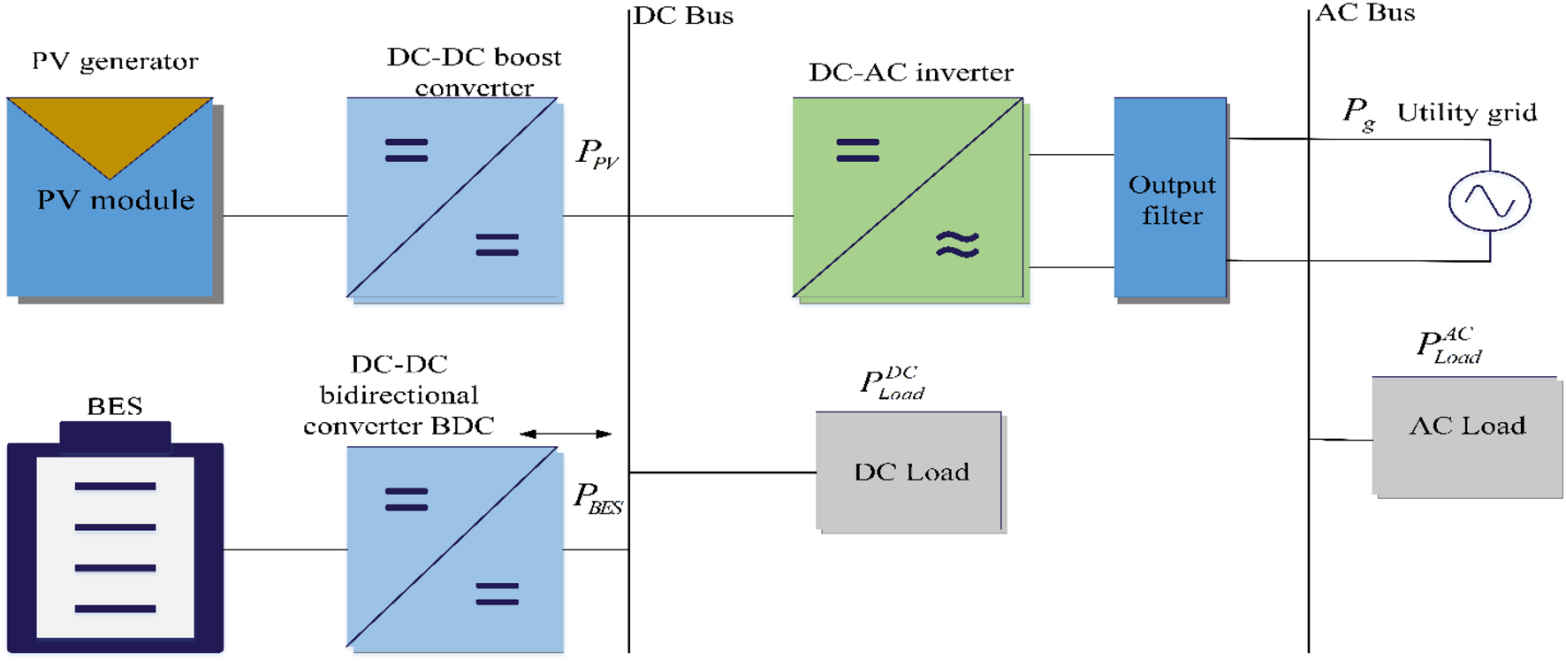
The block diagram of the proposed PV/BES grid-connected system.

In this part, a PV/BES grid-connected system is presented to remove the previously discussed trade-off and minimize DC-Bus capacitance. The proposed PV/BES grid-connected system is described in Fig. 3 as a block diagram. The subsections below discuses several aspects of this diagram.

### DC/DC (step-up) boost converter control

**Figure 4:**
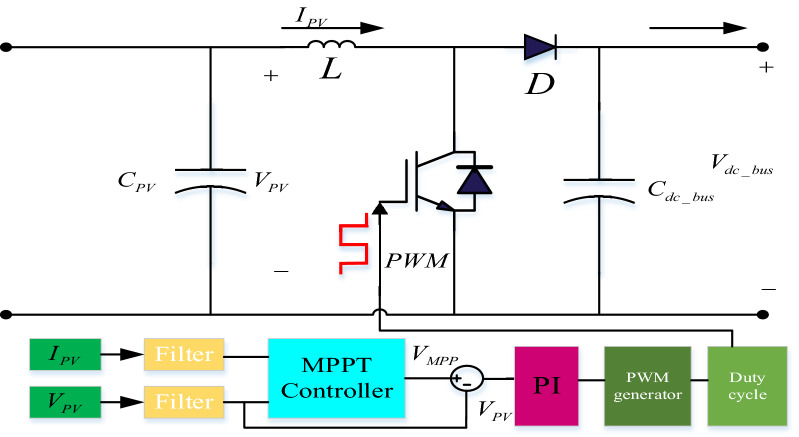
DC/DC boost converter with MPPT tracking.

The DC/DC step-up converter is controlled to provide maximum power extraction from the PV system. The MPPT method is attained based on modifying the incremental conductance (IC) algorithm^27–30^, which uses a PI controller to modify the duty ratio of the step-up converter such that the output power of PV system consistently follows the MPPT-generated reference value. The overall step-up converter circuit and control blocks are shown in Fig. 4.

The slope of the PV curve is detected using the IC technique. The global maximum power point (GMPP) was tracked by looking for the PV curve’s peak. In this MPPT method, the instantaneous conductance (I/V) and incremental conductance (dI/dV) are used. These two quantities are used by the MPPT method to locate the operating point of the PV array in the PV curve. Equation (15) describes the PV array operation at MPP. Consequently, Eq. (16) indicates that the PV array is operating on the left side of the MPP; and Eq. (17) indicates that it is on the right side of the MPP^31–35^.

15 $$\frac{dI}{{dV}} = - \frac{I}{V}$$

16 $$\frac{dI}{{dV}} > - \frac{I}{V}$$

17 $$\frac{dI}{{dV}} < - \frac{I}{V}$$

The preceding equations are derived from the idea that the slope at MPP is zero of the PV curve, as demonstrated in Eq. (18)

18 $$\frac{dP}{{dV}} = 0$$

The following equations can be found by rewriting Eq. (18).

19 $$\frac{dP}{{dV}} = I\frac{dV}{{dV}} + \frac{dI}{{dV}}$$

20 $$\frac{dP}{{dV}} = I + V\frac{dI}{{dV}}$$

21 $$I + V\frac{dI}{{dV}} = 0$$

**Figure 5:**
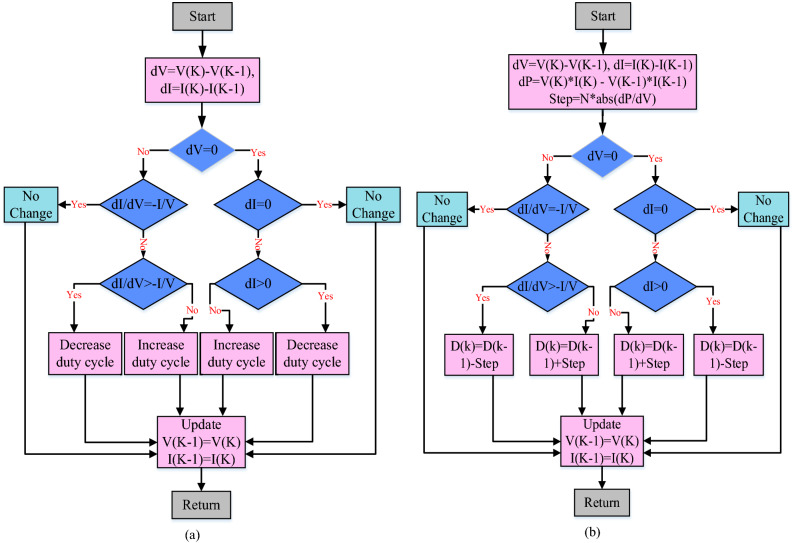
Flowcharts for (**a**) TIC algorithm and (**b**) VSZIC algorithm.

Equation (21) is used to identify the MPP in the TIC approach, and the Flowchart of the TIC technique is illustrated in Fig. 5(a). The sensors of the MPPT controller are the output PV voltage and current. If Eq. (16) is fulfilled, the converter’s duty cycle should be reduced, and vice versa if Eq. (17) is met. The duty cycle remains unchanged if Eq. (21) is fulfilled.

The VSZIC approach developed in^31^ can improve the MPPT controller’s tracking speed. Figure 5(b) depicts the VSZIC algorithm’s flowchart. The algorithm sequences are generally comparable to standard incremental conductance, with the exception of the step size computation. The variable step size approach uses Eq. (22) to change the step size of the duty cycle, where N is the scaling factor.

22 $$Step = N^{*} abs(dP/dV)$$

#### The weakness of TIC and VSZIC

The IC approach detects the MPP by looking at the slope of the PV curve. If the algorithm indicates that the operational point is at the top of the P-V curve (slope = 0) and Eq. (18) is fulfilled, then the DC–DC converter's duty cycle is fixed, and no fluctuation happens until the slope variations. However, as highlighted in^36,37^, the zero slope criteria is seldom met in reality due to numerical differentiation truncation error.

**Figure 6:**
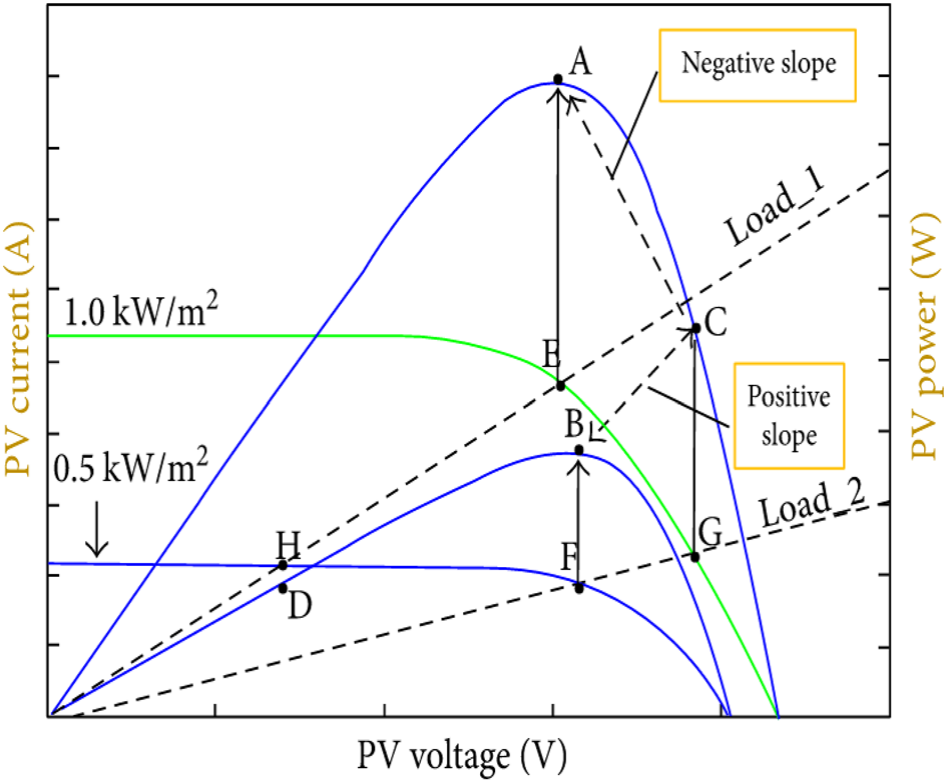
The P–V and I-V curves under radiation 1000 W/m^2^ and 500 W/m^2^.

Apart from steady-state oscillation, when the sun irradiation rises, the TIC algorithm becomes confused. When the irradiance is 500 W/m^2^, the MPPT method changes the duty cycle such that the PV system works at load-2 line and the maximum power point (point B) is fully tracked, as illustrated in Fig. 6. If the sun irradiation rises with time, but the duty cycle remains at load-2 line, load-2 line will intersect with the 1000 W/m^2^ I-V curve at (point G), which matches to the power at (point C) in the P-V curve. The gradient between (points B and C) is determined using the TIC approach which gives a positive gradient, and the power obtained by load-2 line is at (point C). The gradient between (point C) and the 1000 W/m2 MPP (point A) is negative. Therefore, the usual incremental conductance technique responds to new gradient and rises the voltage of the PV module without realizing this inaccuracy. As a result, when the sun radiation levels vary from low to high, the standard method implements an erroneous initial step change. When the sun radiation level drops from high to low, nevertheless, this problematic does not arise. The reason for this is that the gradient is positive from (points E to H), or from (points A to D) on the P–V curve. (Points B and D) have a positive gradient as well.

#### Proposed modified incremental conductance (MIC) algorithm

The slope of the P-V curve, which is influenced by load resistance and solar radiation, is used in the IC method. The PV module’s current and voltage are used in the computation by the algorithm. As a result, the influence of solar radiation and load variations on the PV module’s current and voltage must be carefully addressed in the algorithm.

Table 1 summarizes the variations in the PV module’s voltage and current as a function of solar radiation level and load resistance. Once the PV system is running at (point F) of the load-2 line and solar radiation unexpectedly rises, the PV system’s operating point shifts to (point G), as illustrated in Fig. 6. As a result, both voltage and current rise. On the contrary, once the PV system is running at (point E) of the load-1 line and solar radiation unexpectedly drops, the PV system’s operating point shifts to (point H). As a result, both voltage and current drop. These two sorts of variations are not effectively explored in the TIC technique. In the meantime, if the PV system is operating on load-1 line and the load resistance rises, the PV will be switched to load-2 line, and as a result, the PV panel’s voltage rises while the PV panel’s current falls. When the load resistance falls, the current increases and the voltage decreases.

**Table 1:** The variation of PV panels voltage and current as solar radiation and load resistance variation.

Parameters conditions	Variations of voltage (dV)	Variations of current (dI)
Increase solar radiation	Increase	–
Decrease solar radiation	Decrease	–
Increase load resistance	Increase	Decrease
Decrease load resistance	Decrease	Increase

To remove the steady-state oscillation, a permissible error is used. Equation (21) can be rewritten as:

23 $$I + V\frac{dI}{{dV}} < 0.06$$

With a step size of 0.005 of the duty cycle, the steady-state error in the proposed MIC approach is around (0.7%) using the allowable error of 0.06. Using (1Soltech 1STH-250-WH) PV array, simulation data were gathered to choose the appropriate step size of the duty cycle for the suggested system. When the duty cycle is adjusted at the MPP, three dissimilar types of step sizes were assumed: 0.001, 0.005, and 0.01. However, the MPP may be efficiently tracked in any step size. Among all of the available step sizes, the 0.005 step size demonstrates that the variation in PV module power is the most acceptable, and the controller can respond accurately and quickly when employing it. As a result, the duty cycle step size in the system was set to 0.005. The allowable error in Eq. (23) was set using the lowest slope value. For the 0.005 step size, the minimal slope value is 0.0527. As a result, the allowable error is set at 0.06 to prevent the step size from altering when the slope value is less than this permitted error.

**Figure 7:**
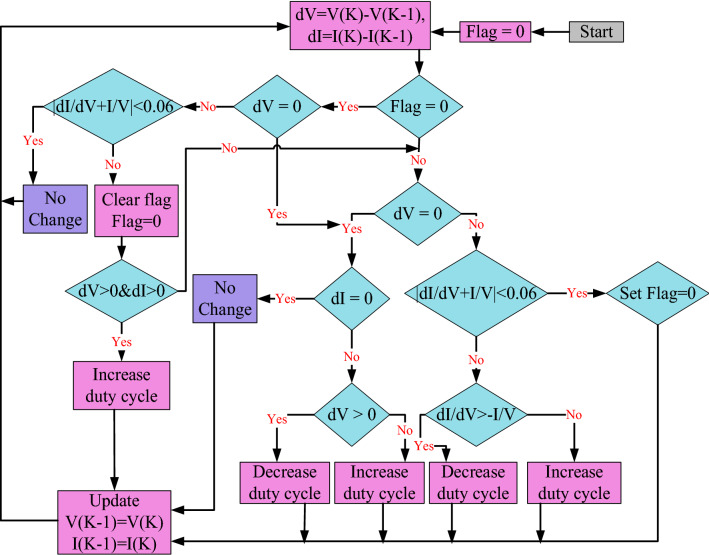
Flowchart of the proposed MIC algorithm.

Finally, the Flowchart of the proposed MIC method is depicted in Fig. 7. A flag value is initially adjusted to be zero. When it sets to 1.0, this flag value shows that the MPP has been achieved. If the flag value is zero, Eq. (23) is used to execute the TIC technique. The system works at the MPP when the criterion in Eq. (23) is met. As a result, the algorithm first sets the flag to 1.0 before proceeding to the enhanced procedure. The program continues to examine the condition of Eq. (23) in the modified procedure. The duty cycle is not changed if the solar irradiance and load resistance stay unchanged. The algorithm sets the flag value to zero when changes in solar irradiation or load occur, and then regulates the variations in the PV module’s voltage and current. If the algorithm detects an increase in both current and voltage, the duty cycle is increased as well. As a result, the IC method has been tweaked to compensate for the erroneous response caused by the increased solar irradiation.

### Flowchart decision logic based control scheme for PV/BES grid-connected inverter

**Figure 8:**
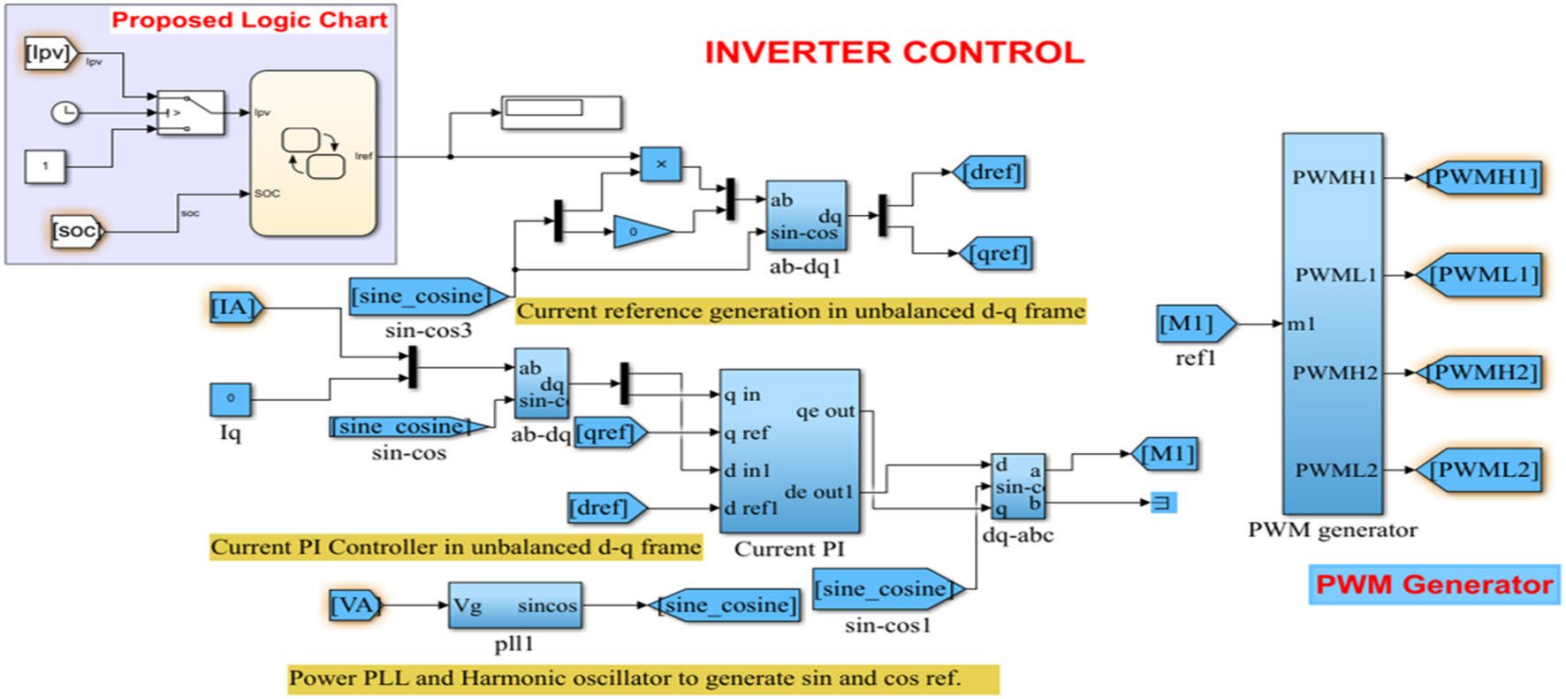
Schematic of proposed d-q control technique.

Figure 8 depicts a diagram of the proposed inverter control structure while Table 2 shows the PI control parameters of the inverter. The grid is connected to the current regulated voltage source inverter. The grid current and voltage are monitored here and sent to the control circuit, which in turn generates the pulse width modulated (PWM) signals required for the current controlled inverter. The sine/cosine signal necessary for Park and Inverse Park transformations is generated using the grid voltage. The feedback signal is calculated from the basic in-phase and quadrature component of grid current, which is then translated to a d-q synchronous reference frame. The PI controller receives the error in d-q currents. Non-interactive control eliminates the influence of voltage loss across the inductance. The PI output, coupling term, and grid voltage make up the whole control signal in the direct axis.

**Table 2:** The PI controller parameters for inverter and DC/DC bidirectional converter.

Inverter		DC/DC bidirectional converter	
Parameters	Tuned	Parameters	Tuned
*Kp*	3	*Kp*	0.00001
*Ki*	27	*Ki*	0.05

**Figure 9:**
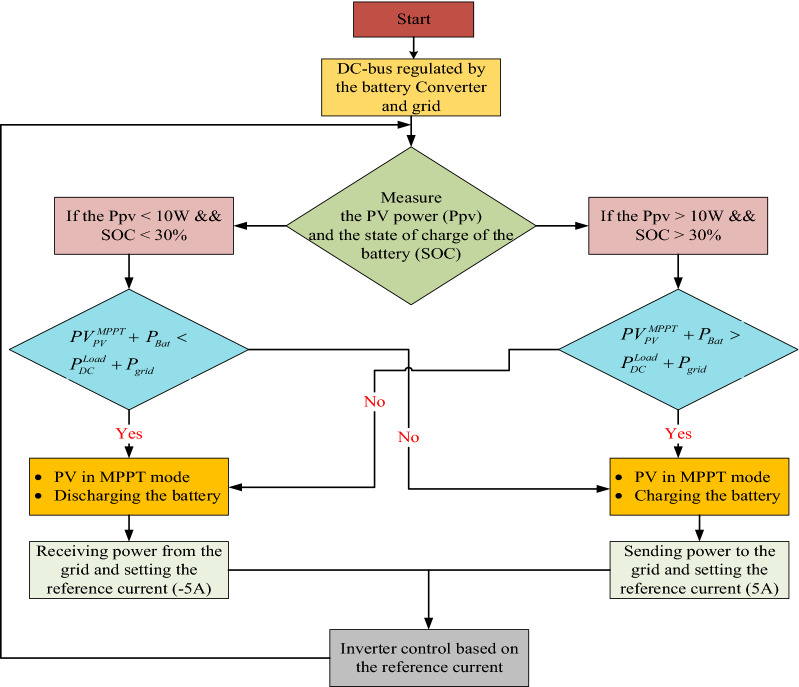
Flowchart of decision logic based control scheme for grid-connected inverter.

As presented in the flowchart of (Fig. 9), some decision logics are implemented to send power from the PV-BES system to the grid via an inverter and LCL filter and also receive power from the grid to the battery and DC load when the PV system is unavailable. Therefore, the power of the PV system and the SOC of the battery were measured and compared with some defined value as shown in Fig. 9.

### The BES bidirectional buck/boost converter control

**Figure 10:**
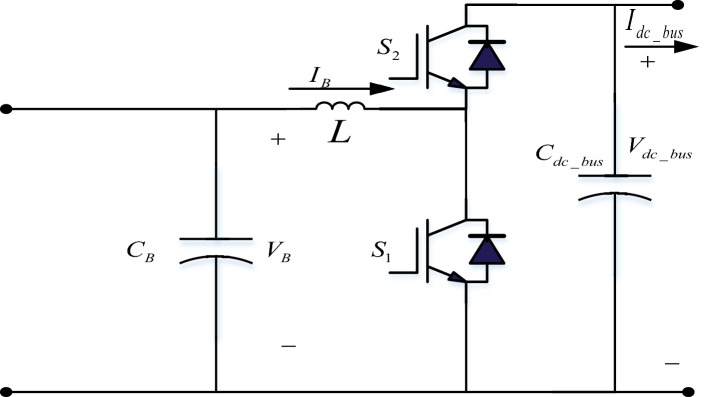
The bidirectional buck/boost converter circuit.

A buck/boost converter circuit with two PI controllers is used to manage the charging/discharging of the BES, as shown in Fig. 10. Table 2 shows the PI control parameters of the bidirectional Buck/Boost converter.

**Figure 11:**
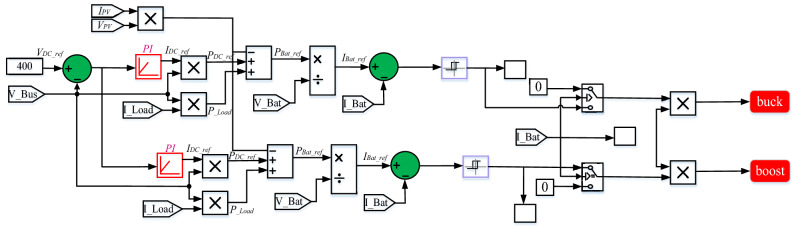
Bidirectional buck/boost converter control.

The DC-Bus voltage error was handled by PI gains, *Kp* and *Ki*, during disturbances at the DC-Bus such that the DC-Bus voltage followed the voltage set point (*V*_*dc-ref*_ = 400 V), as seen in the Fig. 11. The PWM generation circuit receives the output signal from the PI controller, which is then utilized to decide between buck and boost mode of operation. To engross power from the DC-Bus, switch S_1_ is activated and S_2_ is deactivated during step-up discharge mode, whereas S_2_ is triggered and S1 is deactivated during step-down charge mode.

The control current *I*_*dc*_ that flows to the capacitor *C*_*dc_bus*_ is adjusted to its reference current *I*_*dc_Ref*_ in order to regulate the DC-Bus voltage *V*_*dc*_ to the desired value *V*_*dc_busRef*_. According to Fig. 11, the outer voltage loop's PI controller is used to acquire the voltage control.

## Results and discussion

A case study was conducted in this work to prove the effectiveness and robustness of the suggested strategy utilizing MATLAB/ Simulink. The components of the suggested hybrid approach include a PV module, a DC-DC step-up converter, a DC-Bus, a BES and its bidirectional DC-DC converter, an inverter controller, and load modelling. The PV module's job is to provide electricity to the load, while the BES's job is to charge and discharge to store and release energy. Figure 3 depicts a block schematic of the proposed system.

Supplementary Information below has more details on the detailed models. The simulations are run under the next testing conditions to further demonstrate the performance of the proposed methods: first, the proposed MIC tested comparing to the TIC and VSZIC methods in term of steady state oscillations and tracking speed).second, the proposed MIC tested compared the meta heuristic algorithms as well as P&O in term of : (1) temperature fixed at 25 °C and the variation of solar radiation; (2) the solar radiation fixed at 1000 W/m^2^ and variation temperature; (3) constant temperature and radiation with variation in load current.

### Performance of the proposed MIC, TIC and VSZIC methods during steady state oscillations and tracking speed

**Figure 12:**
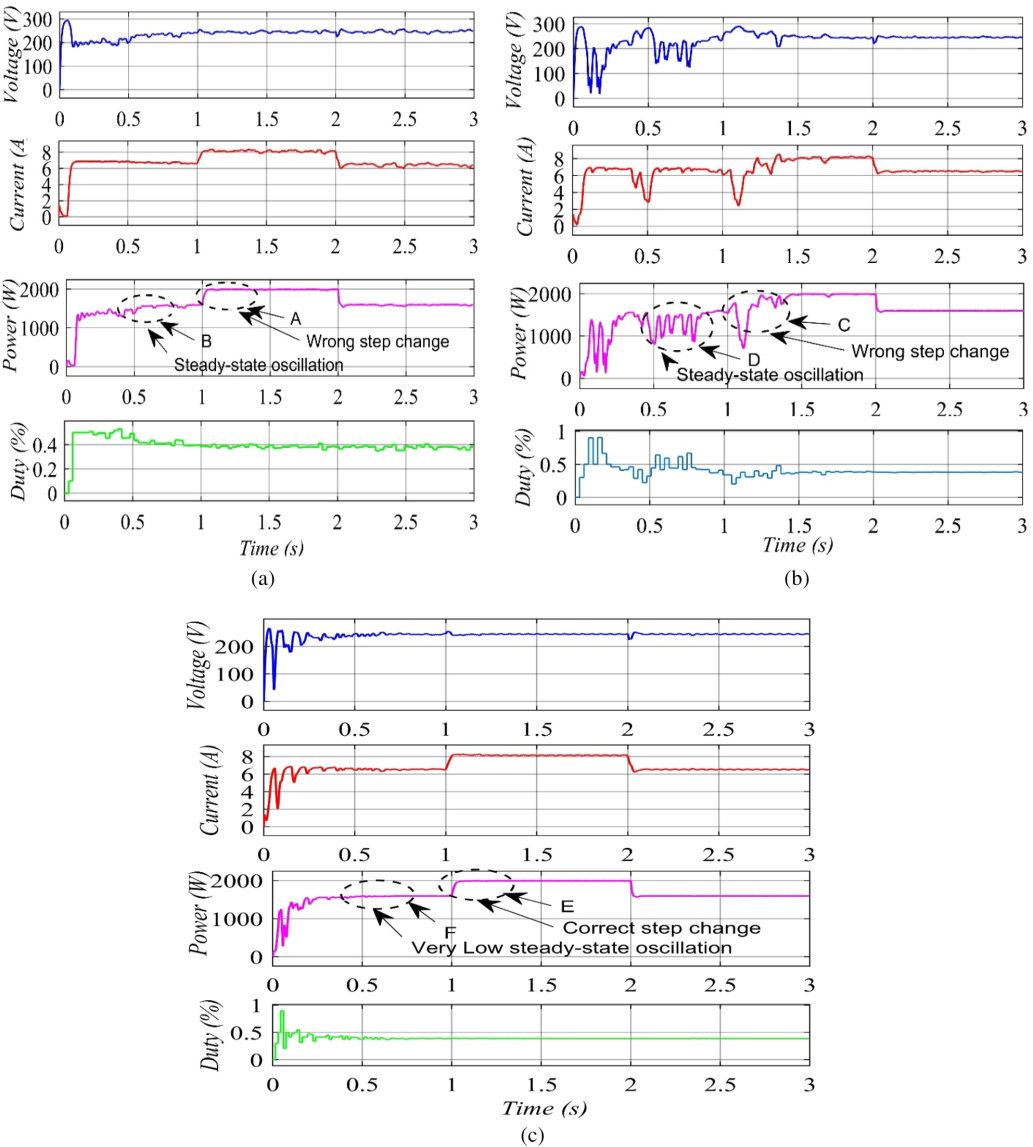
Simulation results: (**a**) TIC algorithm. (**b**) VSZIC algorithm. (**c**) Proposed MIC algorithm.

In order to asses the performance of the three methodologies and compare their differences, simulation is carried out at different low and high solar radiation levels. The variable step size's scaling factor, N, is 0.03 and the MPPT controller's sampling time is 0.05 s. The TIC and MIC algorithms use duty cycle steps of 0.005 for the converter. The simulation results of the TIC  approach are shown in Fig. 12(a). The radiation is set at 800 W/m^2^ at the begining of the simulation, and the MPP is attained at t = 0.6 s. The duty cycle varies from 0.38 to 0.4. The PV module's power (1555–1560 W) and oscillates at the same time. The radiation is raised to 1000 W/m^2^ at time t = 1 s, while the duty cycle stays at 0.38. As a result, the PV module's power rises. The TIC algorithm samples the MPPT controller at time t = 1.75 s. The first step shift in the duty cycle is unclear and elicits incorrect reactions. As a result, the PV module's power is reduced, as indicated by point A in Fig. 12(a). The algorithm then reverses its course and boosts the PV module's power. The MPP for solar irradiation of 1000 W/m^2^ is obtained at t = 1.5 s. The power of the PV module oscillates between (1992 and 1924 W) once more. When the radiation drops to 800 W/m^2^ at time t = 2 s, the TIC method performs as expected.

Results of the VSZIC are displayed in Fig. 12(b). The MPP is attained at t = 0.9 s when the radiation is adjusted to 1000 W/m^2^. The duty cycle varies between 0.5 and 0.6. Because Eq. (21) is used in the method, the duty cycle changes get less, as the operating point gets closer to the peak. As a result, it is also discovered that as time passes, the fluctuation in the power of the PV module (1550–1560 W, point D) gets reduced. The radiation rises to 1000 W/m^2^ at t = 1 s. The MPPT controller, as seen in Fig. 12(b), point C, experiences the same issue that the VSZIC algorithm does with the inaccuracies in duty cycle adjustments. This is a result of the algorithm sequence utilized during the variation in radiation, which is comparable to the TIC method. The power of the PV module oscillates while it is operating in steady state around (1596–1598 W). When the radiation intensity lowers to 800 W/m^2^, it can respond precisely and even more quickly due to the algorithm's use of varying step sizes.

The proposed MIC algorithm's simulation results are shown in Fig. 12(c). The MPP for 800 W/m^2^ is obtained and the duty cycle is kept at 0.388 at t = 0.3 s. The PV module's power is kept constant at 1600 W. Because of the decreased steady-state oscillation, the power losses are smaller. The radiation increases to 1000 W/m2 at time t = 1 s. The rise in radiation is then accurately detected by the suggested technique at t = 1.042 s, as seen in Fig. 12(c), point E. As a result, the power is raised from the first step until the MPP is attained at t = 1.042 s, and the power of the PV module is fixed at 2000 W. The MPP can be reached by the proposed MIC method in just four stages. When compared to the TIC and VSZIC algorithms, which require six steps and five steps respectively, the proposed algorithm responds to changes in radiation more quickly. When the radiation level increases, the proposed MIC method is 0.1 s quicker than the TIC algorithm. Furthermore, when the allowable error approaches 0.06, the steady-state oscillation is minimized.

The proposed method reaches the MPP in four steps. Also, it responds more quickly to changes in solar irradiance than the traditional and variable step size algorithms, which need six and five steps, respectively. During an increase in sun irradiance, the proposed algorithm converged 0.1 s quicker than the conventional algorithm. In addition, the steady-state oscillation is diminished when the permitted error reaches 0.06. Lastly, the computational time required by the proposed algorithm is only 4 instructions longer than the conventional algorithm, which are (1) initialize the flag value to zero, (2) check the flag value is equal to one or zero, (3) clear the flag value if the permitted error is not met, and (4) check the variation in both the current and voltage of PV modules. The proposed method detects the direction of fluctuation in voltage and current before raising or reducing the duty cycle at the next sample time, directly after changes in solar irradiance. In the meanwhile, the traditional algorithm responds instantly following the variation in sun irradiance at the subsequent sampling time.

The accurate response time for a sharp radiation rise in order to catch the global peak (GP) can be seen in the Fig. 12 and Table 3. The proposed method responds accurately and faster when the solar irradiation level increases. Moreover, the proposed algorithm shows zero oscillation in the power of the solar module after the maximum power point (MPP) is tracked by using a small permitted error.

**Table 3:** Experimental performance during radiation patterns for MIC with TIC and VSZIC algorithms.

Overall performance		Time interval			Average of responding time (s)
		Pattern_1	Pattern_2	Pattern_3	
		0–1 s	1–2 s	2–3 s	
Power at GP (W)		1596	2000	1596	
Responding Time (s)	TIC	0.92 s	1.2 s	2.09 s	1.403 s
	VSZIC	0.93 s	1.454 s	2.12 s	1.501 s
	MIC	0.2597 s	1.03 s	2.05 s	1.113 s

### Irradiance variation effect

**Figure 13:**
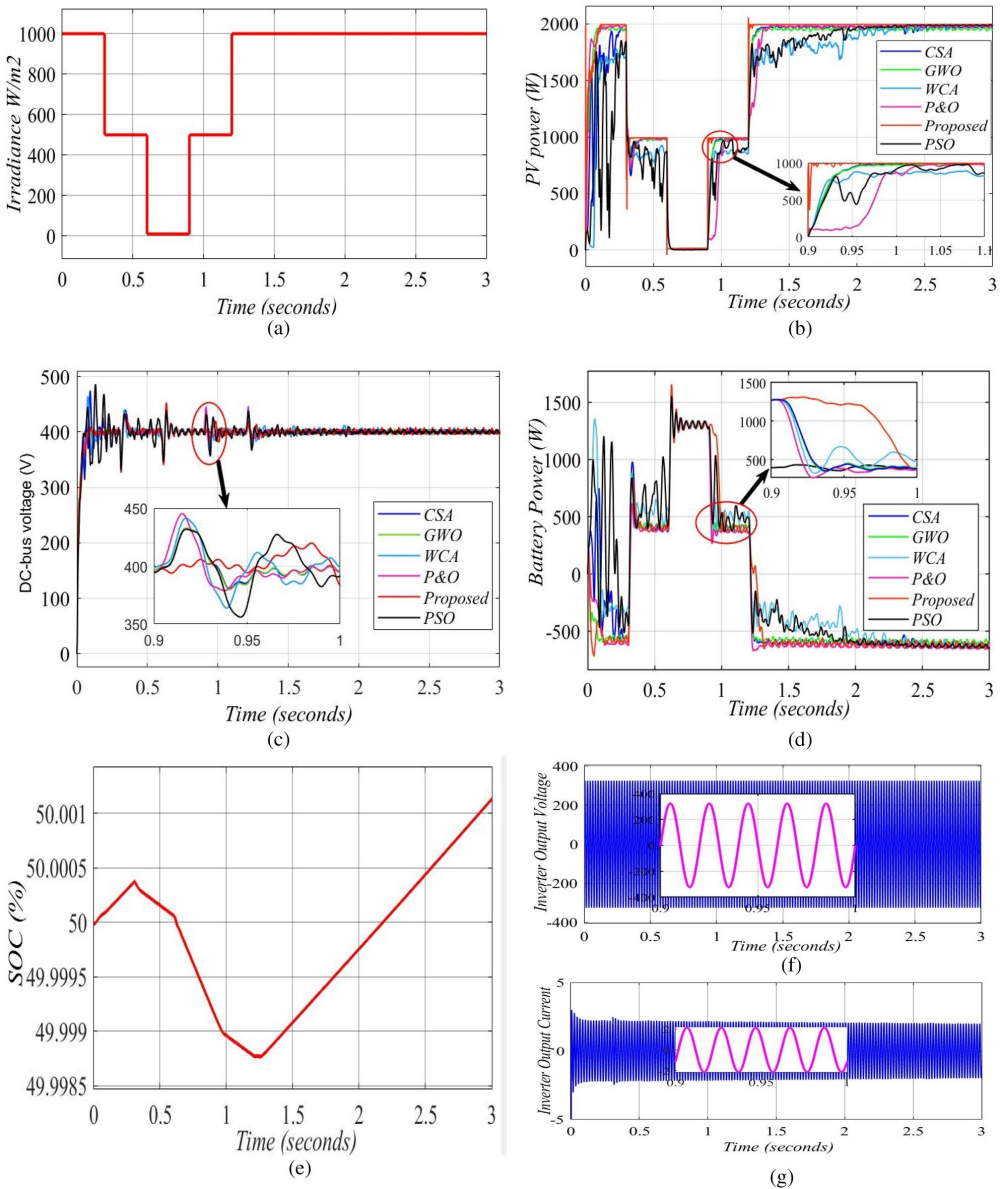
Simulation output of radiation changes in case 1: (**a**) irradiance; (**b**) PV system output power; (**c**) DC-Bus voltage output; (**d**) battery power; (**e**) state of charge (%); (**f**) inverter output-voltage; (**g**) inverter current.

In this scenario, the irradiance is raised between 0.9 and 3 s and dropped between 0.3 and 0.9 s to measure the effectiveness of the hybrid PV/BES system, as seen in Fig. 13(a). The temperature of the PV cell is maintained at 25 °C. At these operating conditions, Fig. 13(b) illustrates the robustness and performance of the proposed MIC-MPPT in terms of PV output power when exposed to a fast variation in solar irradiation. The proposed MIC technique, as shown in Fig. 13(b), can offer greater dynamic operation, quicker convergence, less operating point fluctuations at MPP, and better GP tracking capability GP under varied conditions more successfully than P&O, CSA, PSO, GWO, and WCA. Under rapidly changing atmospheric conditions, the operating point will not deviate too far from MPP, and it will be more effective and robustness.

The efficiency of MPPT approaches is assessed qualitatively using simulations, taking into account the system's steady state response of the output DC-Bus voltage as shown in Fig. 13(c). Aside from the proposed MIC algorithm and d-q current control for a single-phase inverter which are using flowchart decision logic for both the PV power system and the SOC of the battery storage system, the designed control circuit for charging/discharging of BES is achieved using a buck/boost converter with a DC-Bus capacitance of (10 µF). The bus voltage overshoot and undershoot are limited to (1 V) and (2.5 V), respectively, and the THD of the output current is reduced to less than 5%. When utilizing a traditional PI controller, it is simply not possible to use thin film capacitors on the DC-Bus. Therefore, the MIC is used in the DC-Bus control system to stop dual frequency ripples from reaching the output current control system, thus reducing the output current THD. The use of flowchart decision logic for d-q current regulation for a single-phase inverter is presented in this work to decrease DC-Bus voltage overshoot and undershoot. Because of applying the BES’s intended charging/discharging management, the dynamic performance is significantly improved without compromising grid current. Finally, employing the proposed technique, it is simply possible to use thin film capacitors on the DC-Bus.

When the PV system is unable to supply all of the load demand, such as during solar fluctuations or transitory times, the utility grid is used as a backup source, and the BES bank is used as a short-duration power source to meet load need. It is worth noting that, as shown in Fig. 13(d), when solar irradiation drops below a certain level, the battery is drained to give power to the local DC load and the grid, and its SOC drops (see Fig. 13e). In the various irradiance settings, Table 4 compares the real power levels to the proposed methodologies. In addition, as illustrated in Fig. 13(f,g), the grid inverter’s output voltage and current are pure sinewaves.

**Table 4:** The output power of solar modules at changes radiation conditions.

Time (s)	Actual power value (W)	MIC (W)	CSA (W)	GWO (W)	WCA (W)	P&O (W)	PSO (W)
0–0.3	2000	1996	1955	1919	1780	1985	1885
0.3–0.6	1000	995	991	931	930	989	966
0.6–0.9	20	17	6	10	5	14	6
0.9–1.2	1000	995	986	933	890	977	979
1.2–3	2000	1996	1982	1918	1990	1983	1990

### Temperature variation effect

**Figure 14:**
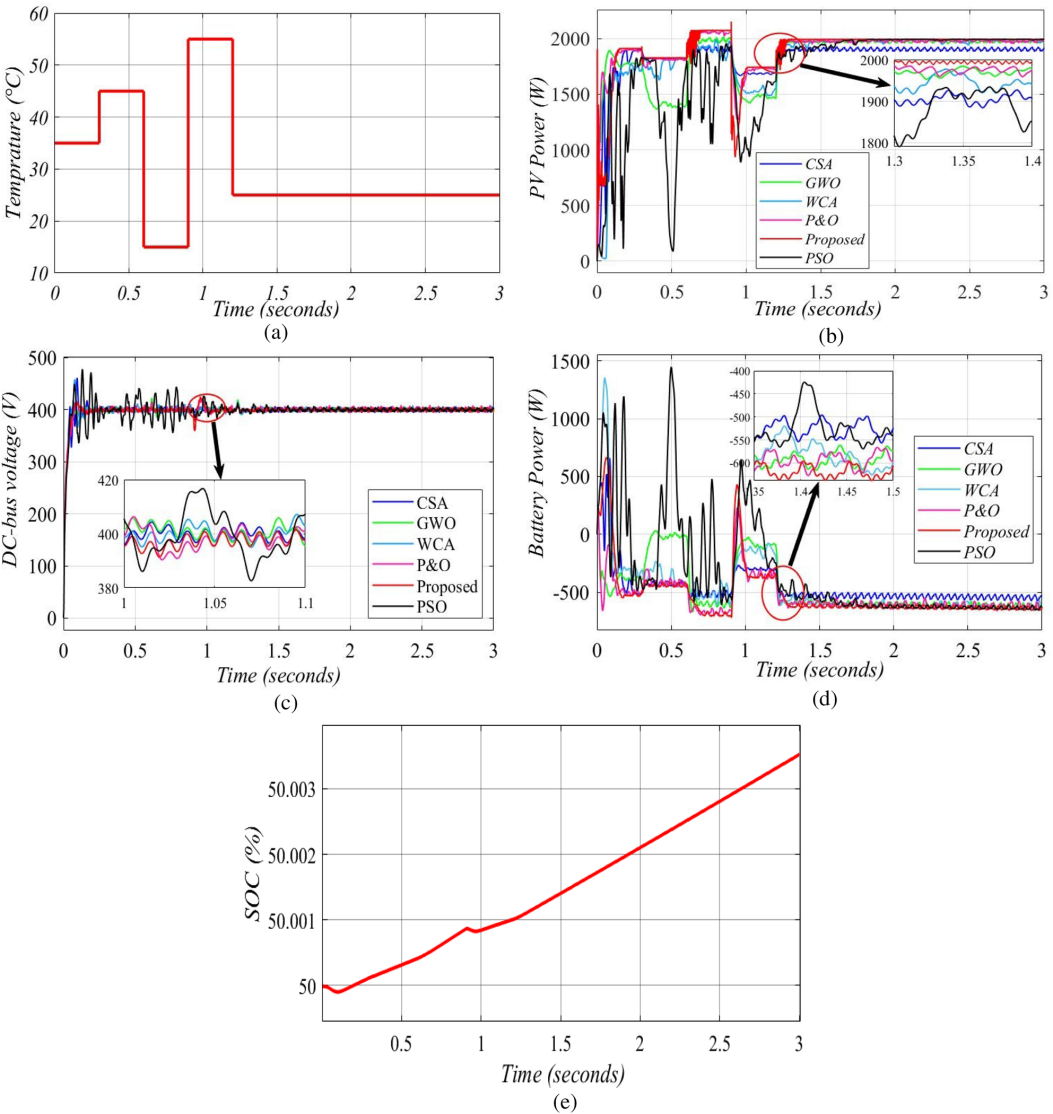
Simulation output of temperature changes in case 2: (**a**) temperature scenarios; (**b**) PV system output power; (**c**) DC-bus voltage output; (**d**) battery output power; (**e**) the SOC % of the battery.

Figure 14(a) shows the influence of temperature fluctuations on the development of the PV array's operating point at a fixed radiation of 1000 W/m^2^. As demonstrated in Fig. 14(b), the proposed MIC algorithm outperforms the P&O, CSA, PSO, GWO, and WCA approaches in terms of tracking the system's MPP. When the cell temperature rises from 35 to 45 °C and from 15 to 55 °C, the MIC algorithm follows MPP softly with minor oscillations, compared to the mentioned techniques. Other MPPT approaches, on the other hand, deviate from the MPP during this temperature increase. The effectiveness of proposed MPPT approaches is assessed qualitatively based on simulations, taking into account the system's steady state response in the DC-bus voltage output, as illustrated in Fig. 14(c). When the PV array has a big value, the battery is charged to give power to the local DC load and the grid when the PV array is not available, as illustrated in Fig. 14(d), and its SOC increases as shown in Fig. 14(e). The inverter's output current and voltage are identical to those shown in Fig. 13(f,g). Table 5 compares real power outputs to the methodologies described under various temperature settings.

**Table 5:** The output power values of solar array in various temperature conditions.

Time (s)	Actual power value (W)	MIC (W)	CSA (W)	GWO (W)	WCA (W)	P&O (W)	PSO (W)
0–0.3	1920	1914	1901	1782	1804	1904	1836
0.3–0.6	1830	1827	1824	1436	1823	1812	1461
0.6–0.9	2080	2077	1934	2038	1991	2064	1956
0.9–1.2	1750	1745	1692	1502	1583	1738	1297
1.2–3	2000	1996	1926	1982	1995	1981	1992

### Load variation effect

**Figure 15:**
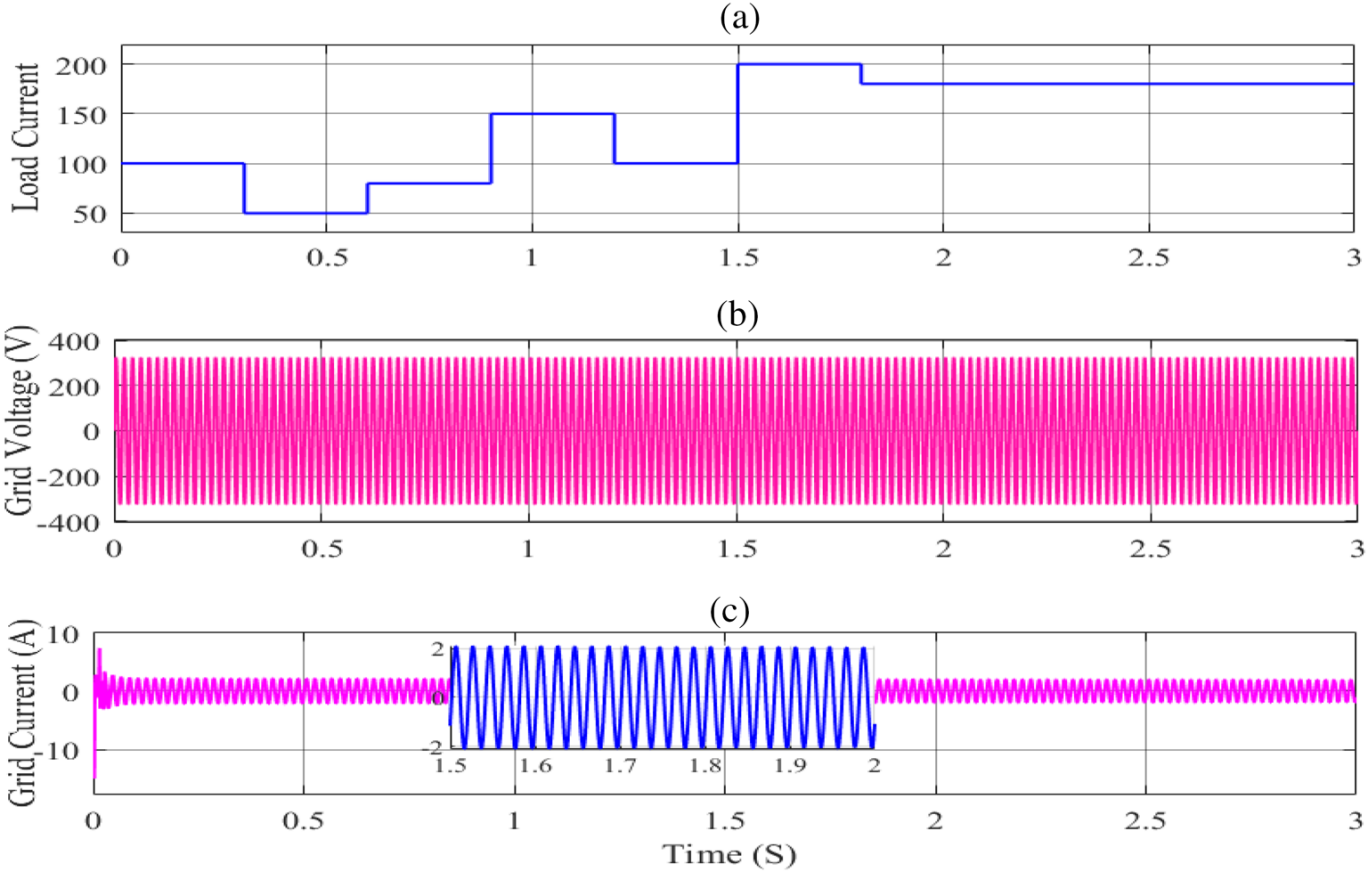
The Current load and AC load output under load changes. (**a**) the load current curve. (**b**) grid voltage and (**c**) grid current.

**Figure 16:**
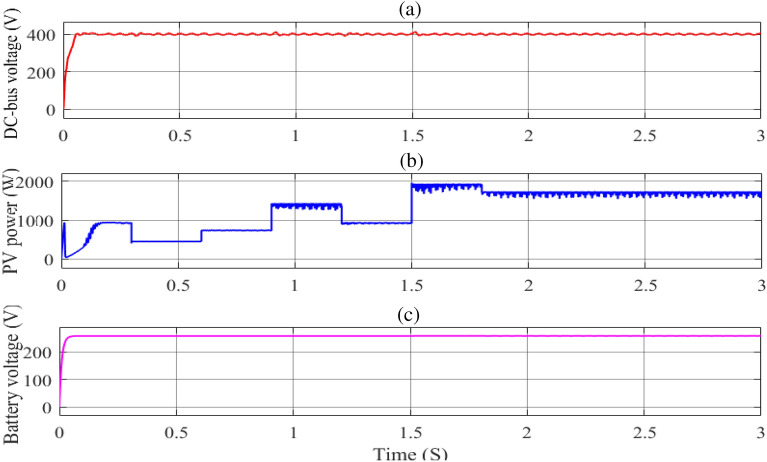
The DC-Bus voltage output under load changes; (**a**) DC-Bus voltage; (**b**) PV power output; (**c**) battery voltage.

Figure 15(a) shows the influence of load fluctuations on the development of the PV array's operating point at a fixed radiation of 1000 W/m^2^ and a temperature of 25 °C. It is worth noting that, as illustrated in Fig. 16(b), the proposed MIC algorithm has improved performance in tracking the system's MPP. A unique approach for managing various elements of the system is suggested, which aids in adjusting the DC-Bus voltage in the event of abrupt load variations while also keeping the system more stable. The DC-Bus voltage under load variations was investigated to assess the PV array controller's robustness. In addition, since there is a small DC-Bus capacitor in the DC-Bus voltage, efforts will be made to find a feasible MIC solution to stabilize the DC-Bus voltage and minimize the power loss caused by photovoltaic voltage fluctuation in PV power generation, as well as develop the dynamic performance of the entire system, as shown in Figs.15(b,c) and 16(a,c).

Finally, the resistive load is changed to confirm that the MIC algorithm is working properly. The resistive load was originally set to 100 Ohm, as illustrated in Fig. 15(a), and the MPP was tracked. The resistive load was then reduced to 50 Ohm. The PV module's current is boosted while the voltage is reduced. The PV array works on the PV curve's left side (Fig. 6). The MIC algorithm reduces the duty cycle of the converter so that it may once again follow the MPP. After that, the resistive load is changed to 80 Ohm, resulting in a drop in PV array current and a rise in PV module voltage. The PV module operates on the PV curve's right side (Fig. 6).To verify the resilience of the proposed technique, the algorithm raises the duty cycle till it hits the MPP again, while the resistive load is still varied.

### Experimental Results and Analysis of the Hardware-in-Loop

**Figure 17:**
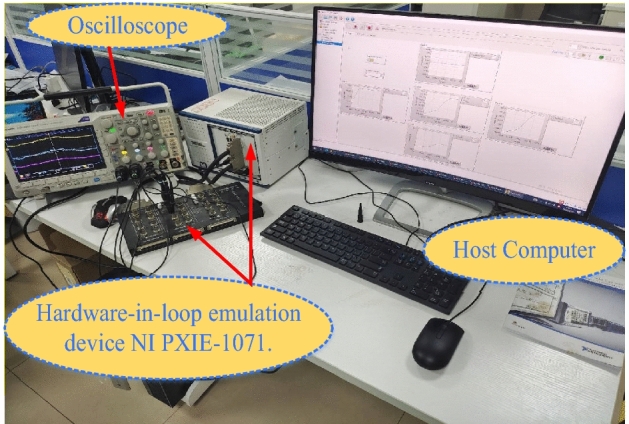
The configuration of Hardware-In-Loop (HIL) experiment.

The experimental platform is set up to evaluate how well the proposed (MIC, TIC and VSZIC algorithms) strategies works. The control method is implemented using NI PXIE-1071. Hardware-In-Loop (HIL) simulation is carried out by the NI PXIE-1071 using HIL software, as shown in Fig. 17. The PV array, boost converter, battery, bidirectional Buck/Boost converter and inverter with LCL filter are the system’s power circuit, which is implemented by the FPGA board of the NI PXIE-1071. Using Simulink's NI standard C language code generation, the sampling and control circuit (MIC, TIC and VSZIC algorithms) based MPPT is converted into a C program and loaded into the NI PXIE-1071control board. The experimental findings are displayed on the power analyzer by connecting an external port board to a group of ports specified in NI PXIE-1071. The sample frequency used is 10 kHz.

**Figure18:**
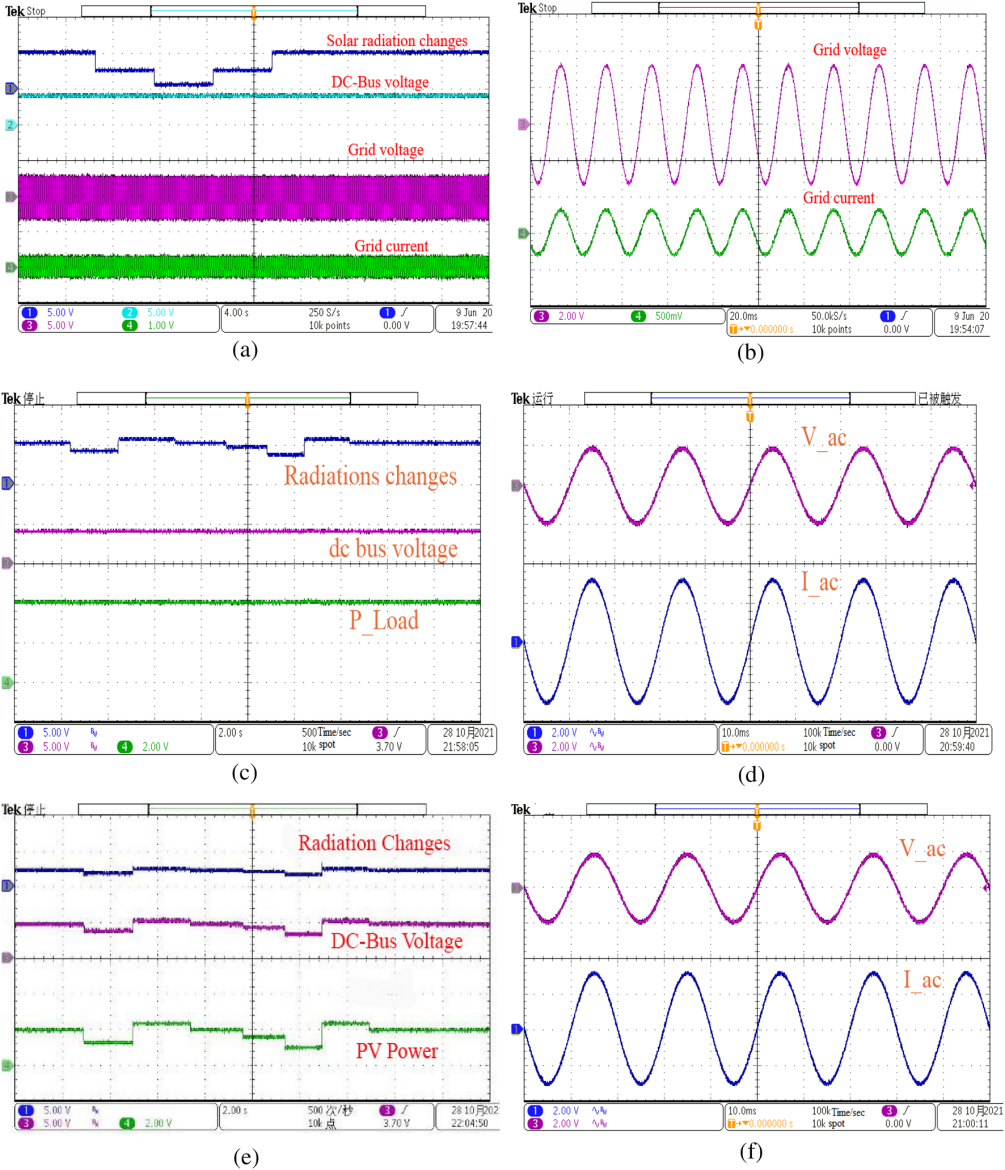
The experimental results of radiations scenarios, AC output, and the DC-Bus voltage output at various radiation levels (**a**, **b**) Proposed MIC; (**c**, **d**) TIC and (**e**, **f**) VSZIC algorithms.

Two alternative scenarios were executed to test the recommended control scheme and model's behavior under various operating conditions, as well as to display the status of the various voltages, currents, power states, and harmonics of the system's components. The first depicts the system's control dynamics performance under different load conditions, while the second depicts dynamic radiation conditions at a constant load. The DC-Bus voltage capacitor's THD is reduced, as shown in Fig. 18. It is a critical component of power quality that should follow the grid rules as well as the nominal frequency design of the load. Even though the system is self-contained, the user must have access to high-quality electricity. The following curves shown in Fig. 18 depict the experimental results under radiation changing situations, demonstrating the higher performance of the proposed control strategy employing MIC compared to TIC and VSZIC algorithms.

In Table 6, the results of the proposed MIC technique is compared to the corresponding results from TIC, VSZIC, and other improved INC techniques proposed in the scientific literature in terms of steady-state oscillation, tracking effectiveness, response time during sudden increases in radiation, and failure of techniques under sudden increases in radiation. In comparison to existing strategies, the proposed MIC technique exhibits a much faster tracking speed, more efficiency, and no reaction to oscillations around the MPP. Therefore, only the proposed MIC algorithm and the technique proposed in^32^ can produce an accurate result under a rapid rise in radiation, and in contrast to the standard approach and those proposed in^33,34^, which produce an inaccurate result.

**Table 6:** The proposed approach comparison to various modified incremental conductance algorithms that have been proposed in published literature.

Technique	Steady-state oscillation	Response time to a sharp rise in radiation	Incorrect choice when radiation suddenly increases	Practical implementation
TIC	Large	Slow	Yes	Yes
VSZIC	Small	Slow	Yes	Yes
Belkaid et al.^38^	Small	Fast	No	Yes
De Brito et al.^39^	Small	Fast	Yes	Yes
Sekhar and Mishra ^40^	Small	Medium	Yes	Yes
Proposed MIC	No	Very fast	Yes	Yes

## Conclusions

One of the main challenges in single-phase PV/BES grid-connected systems is the trade-off between DC-Bus voltage variations, total harmonic distortion (THD) of the output current, and the size of the DC-Bus capacitor. The work presented here investigates this problem and proposes a PV/BES grid-connected system that eliminates this trade-off. The DC-Bus control loop is prepared with the proposed MIC algorithm and d-q current control for this purpose. The proposed MIC algorithm operates to block the harmonics which leads to a low THD in the output current and provides a cost-effective application. Additionally, the d-q current control regulates the DC-Bus stability by controling the PV power and SOC of the battery to eliminate the fluctuations on the DC-Bus voltage. Finally, by avoiding a sudden shift in the energy of the DC-Bus capacitor in the transient reaction, the battery's bidirectional buck/boost converter can adjust the amplitude of the output current in an extremely fast manner. As a result, the dynamic performance is significantly improved while the grid current remains stable. Further, the proposed method is able to decrease the bus voltage overshoot and undershoot up to 97.4% and 90%, respectively, which minimized the DC-Bus capacitor to10-µF and reduced the THD to less than 5%. It was experimentally found that the proposed control of the MIC technique is less susceptible to the harmonic contents of the grid current, and is more robust to the line voltage drop than the conventional strategy.

## Supplementary Information

Supplementary Information.

## Data Availability

The datasets used and/or analysed during the current study available from the corresponding author on reasonable request.
