# Supplementary material for: Maximum power extraction and DC-Bus voltage regulation in grid-connected PV/BES system using modified incremental inductance with a novel inverter control
Source: Sci Rep. 2022 Nov 19;12:19958. doi: 10.1038/s41598-022-22952-0 (PMC9675826; doi:10.1038/s41598-022-22952-0)
Supplement: Supplementary file 1 — Supplementary Information. [file 41598_2022_22952_MOESM1_ESM.docx]

Appendix

1. **Design of the bidirectional buck boost converter of the battery.**

$$\begin{matrix} P=20\times12\times18 \\ V_{in}=20\times12 \\ f_{s}=10e^{3} \\ V_{out}=500 \\ I_{out\_\max}=P/V_{out} \\ del_{IL}=0.1\times I_{out\_\max}\times(V_{out}/V_{in}) \\ del_{V_{out}}=0.1\times V_{out} \\ L=(V_{in}\times(V_{out}-V_{in}))/del_{IL}\times f_{s}\times V_{out} \\ C=(I_{out\_\max}\times(1-(V_{in}/V_{out})))/(f_{s}\times del_{V_{out}}) \\ R=V_{out}/I_{out\_\max} \\ d=(V_{out}-V_{in})/V_{out} \end{matrix}$$

1. **Design of the boost converter for PV system.**

1. **Design of the size of the DC-Bus capacitor.**

$$C_{dc}=\frac{S}{2\omega_{ac}V_{dc}+\tilde{v}_{dc}}$$

1. **Design LCL Calculation.**

$$\begin{matrix} S=2000 \\ V_{dc}=400 \\ F_{sw}=7500 \\ V_{ac}=230 \\ f=50 \\ del_{IL}=0.2\times(S/V_{ac}) \\ L_{1}=(V_{dc}\times(4\times F_{sw}\times del_{IL})) \\ L_{2}=((0.1\times(V_{ac})^{2})(S\times2\times pi\times f))-L_{1} \\ C=(0.5\times S)/((V_{ac})^{2}\times2\times pi\times f) \\ F_{res}=(1/(2\times pi))\times((L_{1}+L_{2})/(L_{1}\times L_{2}\times C))^{1/2} \\ F_{g}=10\times f \\ F_{ss}=0.5\times F_{sw} \end{matrix}$$
